# Safety and tolerability of BAN2401 - a clinical study in Alzheimer’s disease with a protofibril selective Aβ antibody

**DOI:** 10.1186/s13195-016-0181-2

**Published:** 2016-04-06

**Authors:** Veronika Logovinsky, Andrew Satlin, Robert Lai, Chad Swanson, June Kaplow, Gunilla Osswald, Hans Basun, Lars Lannfelt

**Affiliations:** Eisai, Inc., 100 Tice Blvd, Woodcliff Lake, NJ 07677 USA; BioArctic Neuroscience AB, Warfvinges väg 35, 112 51 Stockholm, Sweden; Department of Public Health/Geriatrics, Uppsala University, Dag Hammarskiölds väg 14 B, 751 85 Uppsala, Sweden

**Keywords:** Alzheimer's disease, Amyloid-β, Aβ, Protofibril, Immunotherapy, BAN2401, mAb158, ARIA, Clinical trial

## Abstract

**Background:**

Several monoclonal antibodies for the treatment of Alzheimer’s disease (AD) have been in development over the last decade. BAN2401 is a monoclonal antibody that selectively binds soluble amyloid β (Aβ) protofibrils.

**Methods:**

Here we describe the first clinical study with BAN2401. Safety and tolerability were investigated in mild to moderate AD. A study design was used with staggered parallel single and multiple ascending doses, from 0.1 mg/kg as a single dose to 10 mg/kg biweekly for four months. The presence of amyloid related imaging abnormalities (ARIA, E for edema, H for hemorrhage) was assessed with magnetic resonance imaging (MRI). Cerebrospinal fluid (CSF) and plasma samples were analyzed to investigate pharmacokinetics (PK) and effects on biomarkers.

**Results:**

The incidence of ARIA-E/H on MRI was comparable to that of placebo. BAN2401 exposure was approximately dose proportional, with a serum terminal elimination half-life of ~7 days. Only a slight increase of plasma Aβ_(1-40)_ was observed but there were no measurable effects of BAN2401 on CSF biomarkers. On the basis of these findings Phase 2b efficacy study has been initiated in early AD.

**Conclusions:**

BAN2401 was well-tolerated across all doses. The PK profile has guided us for selecting dose and dose regimens in the ongoing phase 2b study. There was no clear guidance for an effective dose based on biomarkers.

**Trial registration number:**

NCT01230853 ClinicalTrials.gov Registered October 27, 2010.

**Electronic supplementary material:**

The online version of this article (doi:10.1186/s13195-016-0181-2) contains supplementary material, which is available to authorized users.

## Background

Current treatments for Alzheimer’s disease (AD) have no effect on disease progression, which creates a large unmet medical need. Several monoclonal antibodies against amyloid β (Aβ) are in development as potential disease-modifying treatments. Antibodies are attractive drugs as they can be made highly specific for their target. Data from recent AD trials remain inconclusive, but provide some suggestion that a treatment effect of immunotherapy is possible. Bapineuzumab, a monoclonal antibody targeting non-selectively all forms of Aβ, generated side-effects leading to lowering of treatment doses and no treatment effect was found [12]. Solanezumab was developed to target soluble, monomeric Aβ. In two phase 3 studies, solanezumab did not meet primary endpoints [2]. However, when data from the two studies were pooled, a pattern emerged with a slowing of cognitive decline in the subgroup of mild AD. The lack of robust clinical effect with antibodies binding Aβ fibrils or monomers has drawn attention to antibodies targeting soluble, aggregated forms of Aβ.

Aβ exists in various conformational states - monomers, oligomers, protofibrils, and insoluble fibrils [10, 11, 22]. Protofibrils are soluble Aβ aggregates, larger than approximately 100 kDa, i. e., eluting in the void volume on a Size Exclusion Superdex 75 column [1, 8, 9, 19, 20]. There is increasing evidence suggesting that oligomers and protofibrils are more toxic than insoluble fibrils or monomers and that they mediate neurotoxicity and alter synaptic function [7, 10, 21, 22]. The Arctic Alzheimer mutation (AβPP E693G) has been shown to specifically increase the formation of soluble Aβ protofibrils, an Aβ species toxic to neurons and likely to be present in all cases of AD [9]. Indeed, Arctic mutation cases were negative for fibrillar amyloid, as measured by Pittsburg compound B (^11^C-PIB) with positron emission tomography (PET) [13]. This suggests that reducing Aβ protofibrils could provide an effective treatment approach for AD that might ameliorate neuronal toxicity and potentially improve other pathological processes, e.g., synaptic dysfunction, inflammatory changes, and, ultimately, neuronal loss [4, 5, 10].

mAb158 is a murine monoclonal antibody that was raised to target protofibrils [3], and BAN2401 is the humanized IgG1 monoclonal version that selectively binds to Aβ protofibrils. In vitro studies demonstrated that the binding characteristics are essentially indistinguishable from mAb158. BAN2401 has at least a 1000-fold higher selectivity for protofibrils compared to monomers and 10-15 times better binding to protofibrils than to fibrils [14, 15]. Treatment of transgenic mice carrying both the Swedish and the Arctic mutations with mAb158 demonstrated that plaque formation was prevented if the antibody was given before the appearance of plaque in young mice. If treatment started later in this mouse model, levels of insoluble Aβ in the brains of plaque-bearing old mice were not affected. However, in both cases, soluble Aβ protofibril levels were diminished, showing that mAb158 can selectively reduce protofibrils in vivo [6].

The objectives of this study were to evaluate the safety and tolerability of BAN2401 in AD patients following single and multiple ascending doses and to assess the pharmacokinetics (PK) in serum and cerebrospinal fluid (CSF). In addition, effects of BAN2401 on CSF and plasma biomarkers of AD were investigated.

## Methods

### Study design

A multicenter double-blind randomized placebo-controlled study was performed in subjects with mild to moderate AD. No major issues in our animal studies, or in the formal toxicological studies were seen. The starting dose was set with an acceptable margin to NOAEL (No Observed Adverse Effect Level). Estimations of dose levels were made from treatment studies in our transgenic Alzheimer mice models. The top dose was chosen from our studies on transgenic Alzheimer mice and based on lowering of Aβ protofibrils.

**Fig. 1:**
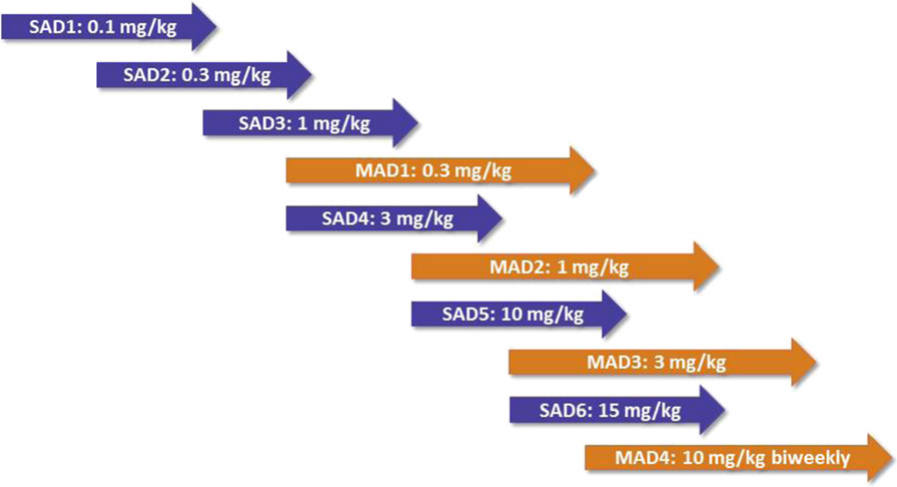
Study design with overlapping SAD-MAD cohorts, where treatment periods took place in a staggered parallel manner. A cohort was initiated after review of the safety and PK data of the previous cohorts. *SAD* single ascending dose, *MAD* multiple ascending dose, *PK* pharmacokinetics

The study was comprised of two parts. In the single ascending dose (SAD) study, doses of 0.1, 0.3, 1, 3, 10, and 15 mg/kg were evaluated. A multiple ascending dose (MAD) part investigated doses of 0.3, 1, and 3 mg/kg administered every four weeks with a total of four doses over four months and a dose of 10 mg/kg biweekly, with a total of seven doses over four months (Fig. 1). Each cohort consisted of eight subjects, two randomized to placebo and six to BAN2401. BAN2401 was given as an i. v. infusion. Treatment periods of the SAD and MAD cohorts took place in a staggered parallel manner. Cohorts were initiated after review of the safety and PK data of the previous cohorts. Before determining to proceed to the next dose level, each SAD cohort was observed for four weeks post dose. This included assessment of a non-contrast brain MRI at three weeks after dosing to evaluate the presence of amyloid related imaging abnormalities (ARIA-E for edema/H for hemorrhage). Treatment in each MAD cohort was initiated after review of all available safety data, including data from the equivalent and the next higher single dose levels. Data included MRI, vital signs, electrocardiogram (ECG) and laboratory data collected at least three weeks post dose in the SAD study. Subjects were followed for up to 180 days after final dosing.

The study was carried out in accordance with the principles of the Declaration of Helsinki and Good Clinical Practice guidelines and was in full compliance with International Conference on Harmonisation guidelines and all applicable local good clinical practice regulations.

### Subjects

Eligible subjects were aged ≥50 years with mild to moderate AD, according to National Institute of Neurological and Communicative Disorders and Stroke and the Alzheimer’s Disease and Related Disorders Association (NINCDS-ADRDA) criteria and Mini Mental State Examination (MMSE) scores of 16-28. Subjects receiving symptomatic treatment for AD were required to have been on stable doses for at least 12 weeks prior to Baseline visit.

### Safety

Safety assessments for SAD, MAD1-3, and MAD4 are outlined in Additional file 1: Addendum, Tables S1A-S3A, respectively. To assess for ARIA, regular non-contrast brain MRI scans were performed to detect ARIA-H [18] according to the schedule outlined in Additional file 1: Tables S1A-S3A. All subjects underwent MRI at baseline. Subsequent MRI scans were performed at three weeks, three months, and at the termination visit at 180 days post dose for SAD1-5 and at 90 days for SAD6. Doses were not administered until evaluation of the most recent MRI. An additional safety MRI was conducted at 90 and 180 days after the final dose for MAD1 and MAD2 cohorts, and at 90 days for MAD3 and MAD4 cohorts.

### Pharmacokinetics

In the SAD cohorts, serum concentrations of BAN2401 were measured pre dose, at 0, 0.5, 1, 2, 4, 8, and 24 h post dose, and at 10, 21, 28, 90 and 180 days post dose. Concentrations of BAN2401 in CSF were measured in SAD6 (15 mg/kg) on day 2 for the first four subjects and on day 10 for the next four subjects. In the MAD cohorts, serum BAN2401 concentrations were measured pre dose and immediately post dose for all four infusions. Additional samples were collected at 0.5, 1, 2, 4, 8, and 24 h post dose after doses 1 and 4. In addition, single samples were collected three weeks and three months post dose. A validated method for measurement of BAN2401 based on liquid chromatography and mass spectrometry (LCMS) (Frontage Laboratories, Exon, PA, USA) was used. The LCMS method had a lower limit of quantification of 0.5 μg/mL. Concentrations of BAN2401 in CSF were measured using a validated ELISA with electrochemiluminence detection with a lower limit of quantification of 3 ng/mL.

### Target engagement and pharmacodynamics

Plasma concentrations of Aβ_(1-40)_ were measured at the same time points as for the PK assessments. It was not possible to measure reliably plasma Aβ_(1-42)_ due to technical difficulties. Evidence for a dose relationship with plasma biomarkers was evaluated. Aβ_(1-42)_, t-tau, and p-tau concentrations were measured in the CSF collected at baseline and in SAD6 and MAD4 10-14 days after the final dose in each cohort. All statistics were descriptive.

### Ethical approval

Ethical approvals were obtained from Copernikus Group IRB, 1 Triangle Drive, #100, Research Triangle Park, NC 27709, Veterans Administration Long Beach Health Care, System IRB, MC 09-151, 5901 East 7th Street, Long Beach, CA 90822, and Research Compliance Administration, IUPUI 980 Indiana Avenue, Room 3315 Indianapolis, IN 46202. Informed consent was obtained from all patients participating in the trial.

## Results

### Subjects

**Fig. 2:**
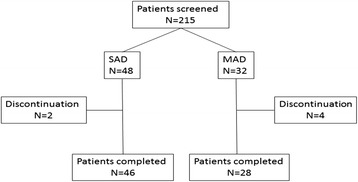
Patient disposition. *SAD* single ascending dose, *MAD* multiple ascending dose

Participant demographics are shown in Table 1. Mean age of the 48 subjects in the SAD cohorts was 70.9 years and mean MMSE score was 23.8. For the 32 subjects in the MAD cohorts, the mean age was 70.0 years and the mean MMSE score was 23.3. Overall, demographic and other baseline characteristics of the groups treated with BAN2401 were similar to those of subjects on placebo. Subject disposition is shown in Fig. 2.

**Table 1:** Subject demographics percentages are based on the total number of subjects with nonmissing values in relevant treatment group

	SAD study								MAD study					
	Placebo	BAN2401 (mg/kg)							Placebo	BAN2401 (mg/kg)				
	(*N* = 12)	0.1 (*N* = 6)	0.3 (*N* = 6)	1 (*N* = 6)	3 (*N* = 6)	10 (*N* = 6)	15 (*N* = 6)	Total (*N* = 36)	(*N* = 8)	0.3 (*N* = 6)	1 (*N* = 6)	3 (*N* = 6)	10 (*N* = 6)	Total (*N* = 24)
Age: mean year (SD)	72.1 (9.2)	70.0 (12.0)	72.7 (6.5)	75.0 (14.0)	68.2 (8.4)	68.7 (9.1)	70.8 (11.5)	70.9 (10.0)	70.0 (11.70)	69.0 (13.19)	69.0 (6.99)	71.8 (11.48)	70.3 (9.81)	70.0 (9.97)
Female gender, n (%)	5 (41.7)	4 (66.7)	2 (33.3)	2 (33.3)	3 (50.0)	2 (33.3)	5 (83.3)	18 (50.0)	2 (25.0)	2 (33.3)	5 (83.3)	4 (66.7)	2 (33.3)	13 (54.2)
MMSE (mean)	23.5	25.2	24.7	23.5	24.0	24.2	21.5	23.8	24.1	24.0	21.7	23.7	23.8	23.3

*MMSE* Mini Mental State Examination, *SAD* single ascending dose, *MAD* multiple ascending dose

### Safety

The numbers of subjects experiencing treatment emergent adverse events (TEAEs) are shown in Tables 2 and 3. There were no trends to suggest an increasing incidence of TEAEs with increasing dose across the SAD cohorts. All of the TEAEs were classified as mild or moderate. The most frequently observed TEAEs with a single dose of BAN2401 were dizziness (8.3 vs. 8.3 % on placebo), fatigue (5.6 vs 8.3 % on placebo), and sinusitis (5.6 vs 0 % on placebo).

**Table 2:** Adverse events in SAD cohorts. Those TEAEs that occurred in more than one cohort in the SAD study are listed

MedDRA Preferred Term	Placebo	BAN2401 (mg/kg)						
	(*N* = 12) (%)	0.1 (*N* = 6) (%)	0.3 (*N* = 6) (%)	1 (*N* = 6) (%)	3 (*N* = 6) (%)	10 (*N* = 6) (%)	15 (*N* = 6) (%)	Total (*N* = 36) (%)
Subjects with any TEAE	8 (66.7)	6 (100.0)	1 (16.7)	3 (50.0)	3 (50.0)	2 (33.3)	5 (83.3)	20 (55.6)
Dizziness	1 (8.3)	2 (33.3)	0	1 (16.7)	0	0	0	3 (8.3)
Fatigue	1 (8.3)	0	0	0	0	0	2 (33.3)	2 (5.6)
Sinusitis	0	1 (16.7)	0	0	1 (16.7)	0	0	2 (5.6)
Asymptomatic ARIA-H	0	0	1 (16.7)	1 (16.7)	0	0	0	2 (5.6)

For each row category, a subject with 2 or more TEAEs with the same PT in that category was counted only once. A TEAE is defined as an AE which started after first dose and within 90 days of last dose.

*AE* adverse event, *MAD* multiple ascending dose, *MedDRA* Medical Dictionary for Regulatory Activities, *PT* preferred term, *SAD* single ascending dose, *TEAE* treatment emergent adverse event

**Table 3:** Adverse events in MAD cohorts. Those TEAEs that occurred in more than one cohort in the MAD study are listed

MedDRA Preferred Term	Placebo	BAN2401 (mg/kg)				
	(*N* = 12) (%)	0.3 (*N* = 6) (%)	1 (*N* = 6) (%)	3 (*N* = 6) (%)	10 (*N* = 6) (%)	Total (*N* = 36) (%)
Subjects with any TEAE	6 (75.0)	4 (66.7)	3 (50.0)	4 (66.7)	4 (66.7)	15 (62.5)
Upper respiratory tract infection	1 (12.5)	2 (33.3)	0	0	2 (33.3)	4 (16.7)
Headache	2 (25.0)	1 (16.7)	0	1 (16.7)	1 (16.7)	3 (12.5)
Orthostatic hypotension	0	1 (16.7)	0	2 (33.3)	0	3 (12.5)
Nausea	0	0	0	1 (16.7)	1 (16.7)	2 (8.3)
Procedural pain	0	1 (16.7)	0	0	1 (16.7)	2 (8.3)
Sinusitis	0	0	1 (16.7)	1 (16.7)	0	2 (8.3)
Somnolence	0	1 (16.7)	0	0	1 (16.7)	2 (8.3)
Urinary tract infection	0	0	0	1 (16.7)	1 (16.7)	2 (8.3)
Vomiting	0	0	0	0	2 (33.3)	2 (8.3)

For each row category, a subject with 2 or more TEAEs with the same PT in that category was counted only once. A TEAE is defined as an AE which started after first dose and within 90 days of last dose.

*AE* adverse event, *MAD* multiple ascending dose, *MedDRA* Medical Dictionary for Regulatory Activities, *PT* preferred term, *SAD* single ascending dose, *TEAE* treatment emergent adverse event

One subject on BAN2401 in the 0.3 mg/kg SAD cohort developed new asymptomatic ARIA-H during treatment. The subject had two microhemorrhages at baseline and developed new asymptomatic ARIA-Hs identified on routine MRI on Days 90 and 180. The subject remained clinically stable throughout the study.

One subject on BAN2401 in the 1 mg/kg SAD cohort experienced an asymptomatic ARIA-H, discovered on Day 21 routine MRI scan considered by the investigator to be possibly related to the study drug. This macrohemorrhage was just above the upper size limit of microhemorrhages. The subject remained asymptomatic throughout the study, and the ARIA-H had completely resolved by Day 180. There was one case initially interpreted as vasogenic edema in a subject on active drug in the 3 mg/kg SAD cohort. However, this was not considered study related as it was associated with a brain nodule secondary to metastasis from a primary lung tumor, present prior to the initiation of treatment.

The most frequently observed TEAEs in subjects treated with multiple doses of BAN2401 were upper respiratory tract infection (16.7 vs. 12.5 % on placebo), headache (12.5 vs. 25 % on placebo), and orthostatic hypotension (12.5 vs. 0 % on placebo) (Tables 2 and 3). All of the TEAEs were classified as mild or moderate with no severe TEAEs.

Six subjects in the MAD cohorts, of which one was on placebo, had ARIA-Hs detected by MRI at baseline. In the 1 mg/kg MAD cohort, new asymptomatic ARIA-Hs emerged during the study in the two subjects on placebo and in one subject on active treatment. The subject on BAN2401 (1 mg/kg) had two new ARIA-Hs identified on MRI after three doses. All three of these subjects remained clinically stable throughout the study. No new abnormal neurological or MRI findings associated with these ARIA-Es were observed.

Over the course of the entire study there were no symptomatic ARIA-Es, ARIA-Es or superficial hemosiderosis seen with either single or multiple BAN2401 doses. No subject experienced a TEAE that resulted in discontinuation or death.

The incidence of treatment-emergent, out-of-range values in hematology, clinical chemistry, or urinalysis parameters was comparable between different doses of BAN2401 and placebo throughout the treatment period. The mean values of vital signs in subjects treated with various BAN2401 doses were generally stable and comparable to those on placebo throughout the treatment period.

### Pharmacokinetics

#### SAD cohorts

**Fig. 3:**
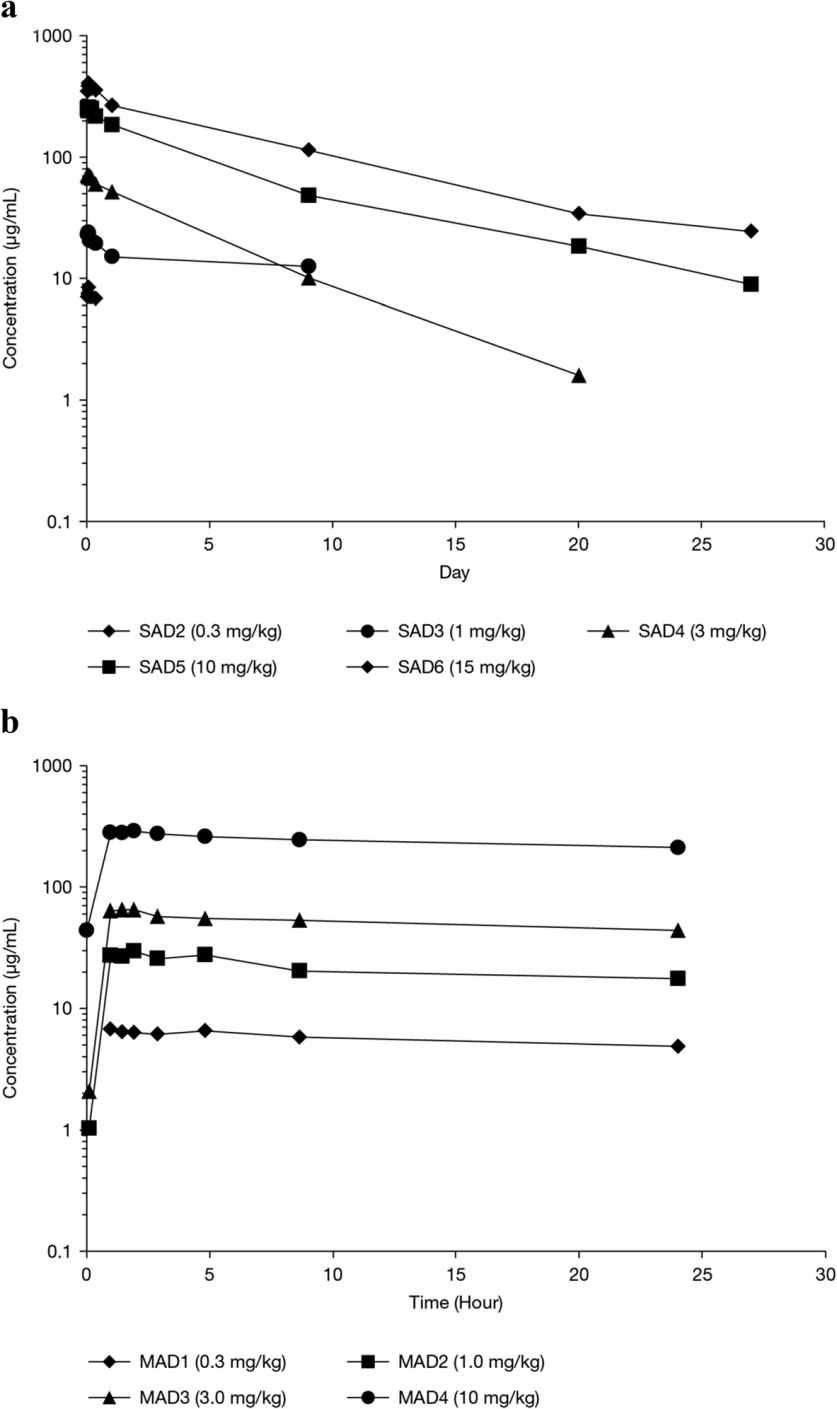
**a** Pharmacokinetics of BAN2401, with mean serum concentration of BAN2401 in SAD cohorts. **b** Mean serum concentration of BAN2401 after the last dose in MAD cohorts of 0.3 – 3 mg/kg every 28 days (4 doses), or 10 mg/kg biweekly (7 doses). *SAD* single ascending dose, *MAD* multiple ascending dose

Mean serum concentration time profiles of BAN2401 for ascending doses are shown in Fig. 3a and findings for the pharmacokinetic parameters are listed in Table 4. The median t_max_ occurred at approximately 1.8 to 2.2 h from the start of infusion. The mean C_max_ and the AUC increased approximately proportionally with BAN2401 dose from 0.3-15 mg/kg. Single BAN2401 doses from 0.3-15 mg/kg showed first order elimination kinetics. Due to limitations in the assay, mean serum half-lives for BAN2401 were only estimated for subjects receiving 10 and 15 mg/kg of BAN2401 and were 165 h (6.9 days) and 174 h (7.3 days), respectively.

**Table 4:** Pharmacokinetic parameters of BAN2401

Cohort	n	Dose (mg/kg)	Cmax (μg/mL)	AUC(0-24) (μg*h/mL)	AUC(0-inf) (μg*h/mL)	t1/2 (h)
SAD1	6	0.1	NC	NC	NC	NC
SAD2	6	0.3	8.50 (2.4)	NC	NC	NC
SAD3	6	1	24.7 (3.6)	NC	NC	NC
SAD4	6	3	74.2 (11)	1390 (140)	7430 (1210)	NC
SAD5	6	10	264 (32.4)	5010 (550)	38000 (7340)	165 (45.5)
SAD6	6	15	418 (54.5)	7630 (593)	66900 (17600)	174 (36.1)
MAD1	6	0.3	7.26 (1.5)	133 (23)	NA	NC
MAD2	3	1	30.6 (4.6)	470 (110)	NA	NC
MAD3	5	3	68.8 (9)	1220 (132)	NA	NC
MAD4	6	10	307 (70.2)	5720(1230)	NA	127(30)

PK parameters for the MAD cohorts were calculated after the final dose of BAN2401. Values reported are Mean (SD)

*AUC* area under the curve, *NC* not calculated due to insufficient data, *NA* not applicable for dosing with multiple doses.

#### MAD cohorts

Mean serum concentration time profiles are shown in Fig. 3a and b and findings for other pharmacokinetic parameters are listed in Table 4. Overall, mean C_max_ and AUC values for the first dose of BAN2401 in each MAD cohort were consistent with corresponding doses in the SAD cohorts. Mean C_max_ and AUC values of the first dose and the final dose increased approximately dose proportionately from 0.3 mg/kg monthly to 10 mg/kg biweekly. The PK profile during the elimination phase was consistent with first-order kinetics. The mean half-life of BAN2401 ranged from approximately 127 h (5.3 days) after the final dose in the 10 mg/kg biweekly MAD cohort, to 174 h (7.3 days) in the 15 mg/kg SAD cohort.

As expected based on the half-life, no accumulation was observed when BAN2401 was administered at 28 day intervals. Biweekly infusions of BAN2401 (10 mg/kg) achieved steady state serum concentrations after the third dose, i.e., after approximately six weeks of treatment. The minimum observed concentration at steady-state (Css, min) achieved with this dosing regimen was approximately 40 μg/mL, with an accumulation factor of approximately 1.4.

#### CSF: serum ratios

Concurrent concentrations of BAN2401 in serum and CSF are shown in Table 5, together with the CSF:serum ratios at 24 h and Day 10-14 after dosing. Due to sampling error only two subjects in the 15 mg/kg SAD group had CSF concentrations measured on Day 10. One subject had a CSF concentration of 624 ng/mL (CSF: serum ratio 0.81 %) and the other a CSF concentration of 72 ng/mL (CSF:serum ratio 0.07 %). In the three lower dose MAD cohorts in which there was no accumulation, the CSF:serum ratios were 0.04 - 0.08 % at 24 h after the final dose (Table 5). However, in the 10 mg/kg biweekly MAD cohort there was BAN2401 accumulation in serum. In this cohort, the CSF:serum ratio was 0.13 % at 24 h after the final dose, and increased to 0.29 % at 14 days after the final dose.

**Table 5:** CSF and corresponding serum concentrations and CSF: serum ratios

	BAN2401 levels 24 h after final dose			BAN2401 levels 10-14 days after final dose		
Cohort (dose mg/kg)	CSF (ng/mL)	Serum (μg/mL)	CSF:serum Ratio	CSF (ng/mL)	Serum (μg/mL)	CSF:serum ratio
SAD6 (15)	96.3 (45.1)	265 (63.1)	0.04 % (0.03 %)	72.2, 624^a^	104 (31.3)	0.07 %, 0.81%^a^
MAD1 (0.3)	3.47 (2.1)	4.85 (1.3)	0.08 % (0.05 %)	CSF not collected		
MAD2 (1)	8.89 (5.31)	17.6 (1.05)	0.04 % (0.03 %)			
MAD3 (3)	25.0 (13.3)	44.1 (4.55)	0.06 % (0.04 %)			
MAD4 (10)	263 (106)	211 (46.0)	0.13 % (0.03 %)	116 (109)	37.5 (21.8)	0.29 % (0.14 %)

Values are expressed as mean and (SD)

*CSF* cerebrospinal fluid, *SAD* single ascending dose, *MAD* multiple ascending dose

^a^Individual values are shown because n = 2

#### Target engagement and pharmacodynamics

**Fig. 4:**
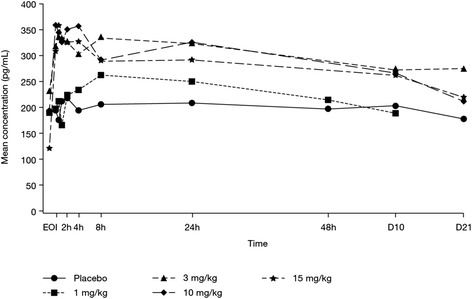
Mean concentrations of Aβ_(1-40)_ versus nominal time for SAD cohorts. *Aβ* amyloid β, *SAD* single ascending dose

BAN2401 was associated with small dose-dependent increases in plasma Aβ_(1-40)_ in the MAD cohorts within a few hours after the first dose as well as the final dose (Fig. 4, Table 6). Plasma Aβ_(1-40)_ levels declined over time with the fall in serum concentration of BAN2401. However, there were no clear effects of BAN2401 on CSF Aβ_(1-42),_ t-tau, or p-tau (data not presented) compared with placebo in the MAD cohorts.

**Table 6:** Plasma pharmacodynamics in MAD cohort

	Placebo	MAD2	MAD3	MAD4
Plasma Aβ(1-40) mean (SD) concentration (pg/mL)				
Maximal % change from baseline	-27.39 (48.98)	15.92 (97.17)	39.71 (17.91)	120.71 (53.21)
24 h post final dose	13.42 (28.09)	16.99^a^	17.50 (9.14)	86.20 (48.06)

Percentage change from baseline in Aβ _(1-40)_

*Aβ* amyloid β, *MAD* multiple ascending dose

^a^Insufficient “n” to calculate SD

## Discussion

Bapineuzumab and other early Aβ immunotherapy programs have encountered safety issues mainly due to ARIA-Es. A beneficial safety profile allowing for efficacious dose levels without undesired side effects is pivotal for success. Safety and tolerability were the primary outcome measures in this first-in-human study with BAN2401 in subjects with mild-to-moderate AD. In this study, BAN2401 was safe and well-tolerated at all doses tested. The maximum tolerated dose was not reached with either single or multiple dosing.

Asymptomatic ARIA-H occurred in 3/60 (5 %) subjects treated with BAN2401, which is a lower incidence than that observed in subjects treated with placebo (2/20, 10 %). The incidence of ARIA in both placebo and BAN2401 treatment groups at base-line was within the expected levels [16]. For the ongoing phase 2b study there were no dosing limitations based on safety, as all doses were safe and tolerable.

Results of the non-compartmentalized PK analyses indicate that BAN2401 is characterized by linear PK, with dose-proportional exposure and first order elimination. The mean serum half-life of BAN2401 was approximately seven days, which was reliably determined when given at doses of 10 mg/kg or higher. This was shorter than predicted from animal PK studies. Consequently, a more frequent biweekly dosing interval was considered important, and an additional MAD cohort with 10 mg/kg biweekly dosing was added. The 10 mg/kg biweekly dose achieved minimum steady state levels of approximately 40 μg/mL after three doses, with an accumulation factor of approximately 1.4. The PK profile has guided us for selecting dose and dose regimens in the ongoing phase 2b study.

BAN2401 penetrated the blood-brain barrier and could be measured in CSF as a surrogate for CNS exposure. Due to the limited numbers of CSF samples, a cautious interpretation of the data is necessary. CSF concentration 24 h after a single dose was 0.04 % of its serum concentration and after monthly multiple doses 0.04 - 0.08 %, but was somewhat higher, 0.13 % at 24 h post dose at steady state after biweekly dosing. Over a 14-day interval at steady state with multiple dosing at 10 mg/kg biweekly, the CSF: serum ratio increased to 0.29 %, which may suggest a longer half-life in CSF vs. plasma.

There were no apparent effects of BAN2401 on CSF t-tau, p-tau or Aβ_(1-42)_. At present there is no validated assay for measuring protofibrils in human CSF. Thus, it was not possible to assess target engagement of BAN2401 in CSF. However, ex vivo immunoprecipitation experiments with BAN2401 demonstrated that the antibody was able to pull-down virtually all Aβ_(1-42)_ from AD brain tissue in the fraction containing mainly soluble Aβ aggregates. This indicates a high level of target engagement in affected brains [17]. There was no clear guidance for an effective dose based on biomarkers. For this reason, several doses and dose regimens are being tested in the ongoing phase 2b study. BAN2401 treatment led to only a slightly increased plasma Aβ_(1-40)_ (Table 6), which was most evident at 10 mg/kg biweekly, and this would be consistent with low affinity of BAN2401 for monomeric Aβ present in human plasma.

Traditionally, SAD and MAD studies involve the completion of each cohort before proceeding to a higher dose. In this study, we used a design allowing the SAD and MAD cohorts to be intercalated and conducted in staggered parallel fashion. Thus, the study duration was significantly shortened. An adequate duration of follow up for the assessment of ARIA and other potential adverse events could still be ensured prior to initiation of higher dose cohorts. In addition, PK results from completed earlier cohorts were used to amend the MAD dose frequency to reflect the shorter than expected half-life and to investigate if the PK profile in CSF might be comparable to that in serum. Use of this innovative study design enabled all cohorts to be completed within 12 months. It demonstrated that in this Phase 1 study BAN2401 was safe and well-tolerated at single doses up to 15 mg/kg and multiple doses up to 10 mg/kg biweekly. On the basis of this study, a Phase 2b efficacy study has been initiated in a combined population of prodromal AD and mild AD dementia.

## Conclusions

Several monoclonal antibodies for the treatment of AD have been in development over the last decade. BAN2401 is a monoclonal antibody that selectively binds soluble Aβ protofibrils. The first clinical study described in this paper demonstrates that that BAN2401 was safe and well tolerated in mild to moderate AD. The presence of ARIA was assessed at multiple time points with MRI. The incidence of ARIA on MRI was comparable to that of placebo. BAN2401 exposure was approximately dose proportional, with a serum terminal elimination half-life of approximately seven days. Only a slight increase of plasma Aβ_(1-40)_ was observed but there were no measurable effects of BAN2401 on CSF biomarkers. On the basis of these findings, a Phase 2b efficacy study has been initiated in early AD.

## Additional file

Additional file 1: Tables addendum. (PDF 125 kb)

## Abbreviations

AD: Alzheimer’s disease
ARIA: Amyloid related imaging abnormalities
AUC: Area under the curve
Aβ: Amyloid β
CSF: Cerebrospinal fluid
MAD: Multiple ascending dose
MRI: Magnetic resonance imaging
PET: Positron emission tomography
PK: Pharmacokinetics
SAD: Single ascending dose
