# Supplementary material for: Safety and tolerability of BAN2401 - a clinical study in Alzheimer’s disease with a protofibril selective Aβ antibody
Source: Alzheimers Res Ther. 2016 Apr 6;8:14. doi: 10.1186/s13195-016-0181-2 (PMC4822297; doi:10.1186/s13195-016-0181-2)
Supplement: Additional file 1: — Tables addendum. (PDF 125 kb) [file 13195_2016_181_MOESM1_ESM.pdf]

Addendum

Table 1A. SAD Safety Procedures

SAD										
**Phase**	**Prerandomization**		**Randomization**							
**Period**	**Screening**	**Baseline**	**Treatment**							
**Visit**	**1**	**2**	**3**		**4**	**5**	**6**	**7**	**8 (Final / Early Withdrawal)**	**Unscheduled (U)**
**Day**	**−60 to −5**	**−1**	**1**	**2**	**10**	**21±2**	**28±2**	**90±5**	**180±5**	
Pre-MRI assessments	X					X		X	X	X
MRI	X[b]					X		X	X	X
Vital signs	X	X	X	X	X	X	X	X	X	X
Physical exam	X	X	X		X	X	X	X	X	X
ECG	X	X	X	X		X	X	X	X	X
Clinical labs (hematology, clinical chemistry, urinalysis)	X	X	X	X	X	X	X	X	X	X
C-SSRS	X	X		X	X	X	X	X	X	X
Adverse events	X	X	X	X	X	X	X	X	X	X

Table 2A. MAD 1-3 Safety Procedures

MAD 1-3													
**Phase**	**Prerandomization**			**Randomization**									
**Period**	**Screening**	**Baseline**		**Treatment**									
**Visit**	**1**	**2**	**3**	**4**		**5**	**6**	**7**	**8**	**9**	**10**	**11**	**12**
**Day**	**−60 to −5**	**-2**	**-1**	**1**	**2**	**21±2**	**28**	**49±2**	**56**	**83**	**84**	**85**	**175±5**
**Procedures/Assessments**													
MRI	X					X		X		X			X
Vital signs	X	X		X	X	X	X	X	X	X	X	X	X
Physical Exam	X	X		X		X	X	X	X	X			X
ECG	X	X		X	X		X		X		X		X[w]
Clinical labs (hematology, clinical chemistry, urinalysis)	X		X	X	X	X	X	X	X		X	X	X
C-SSRS	X	X			X	X	X	X	X		X	X	X
Adverse events	X	X	X	X	X	X	X	X	X	X	X	X	X

Table 3A. MAD4 Safety Procedures

MAD4																				
**Phase**	**Prerandomization**		**Randomization**																	
**Period**	**Screening**	**Baseline**		**Treatment**																
**Visit**	**1**	**2**	**3**	**4**	**5**	**6**	**7**	**8**	**9**	**10**	**11**	**12**	**13**	**14**	**15**	**16**	**17**	**18**	**19 (Final / Early Withdrawal)**	**Unscheduled**
**Day**	**-60 to -5**	**-2**	**-1**	**1/2**	**7**	**14**	**21**	**28**	**35**	**42**	**49**	**56**	**63**	**70**	**77**	**84**	**85**	**98**	**175**	
**Procedures / Assessments**																				
Randomization			X																	
MRI	X						X				X				X				X	X
Vital signs	X	X	X	X	X	X	X	X	X	X	X	X	X	X	X	X	X	X	X	X
Physical exam	X	X		X	X	X	X	X	X	X	X	X	X	X	X	X		X	X	X
ECG	X	X		X		X		X		X		X		X		X	X	X	X	X
Clinical labs (hematology, clinical chemistry, urinalysis)	X		X	X		X		X		X		X		X		X	X	X	X	X
C-SSRS	X	X		X		X		X		X		X		X		X	X	X	X	X
Adverse events	X	X	X	X	X	X	X	X	X	X	X	X	X	X	X	X	X	X	X	X
